# Spectrum of Treg and self-reactive T cells: single cell perspectives from old friend HTLV-1

**DOI:** 10.1093/discim/kyae006

**Published:** 2024-05-13

**Authors:** Masahiro Ono, Yorifumi Satou

**Affiliations:** Department of Life Sciences, Imperial College London, London, United Kingdom; Joint Research Center for Human Retrovirus Infection, Kumamoto University, Kumamoto, Japan; Joint Research Center for Human Retrovirus Infection, Kumamoto University, Kumamoto, Japan

**Keywords:** HTLV-1, regulatory T-cells, self-reactive T-cells, memory-phenotype T-cells, T cell receptor signalling, Nr4a3, Foxp3, Tocky

## Abstract

Despite extensive regulatory T cell (Treg) research, fundamental questions on *in vivo* dynamics remain to be answered. The current study aims to dissect several interwoven concepts in Treg biology, highlighting the ‘self-reactivity’ of Treg and their counterparts, namely naturally-arising memory-phenotype T-cells, as a key mechanism to be exploited by a human retroviral infection. We propose the novel key concept, *Periodic T cell receptor (TCR)-signalled T-cells*, capturing self-reactivity in a quantifiable manner using the Nr4a3-Timer-of-cell-kinetics-and-activity (Tocky) technology. Periodic and brief TCR signals in self-reactive T-cells contrast with acute TCR signals during inflammation. Thus, we propose a new two-axis model for T-cell activation by the two types of TCR signals or antigen recognition, elucidating how Foxp3 expression and acute TCR signals actively regulate Periodic TCR-signalled T-cells. Next, we highlight an underappreciated branch of immunological research on Human T-cell Leukemia Virus type 1 (HTLV-1) that precedes Treg studies, illuminating the missing link between the viral infection, CD25, and Foxp3. Based on evidence by single-cell analysis, we show how the viral infection exploits the regulatory mechanisms for T-cell activation and suggests a potential role of periodic TCR signalling in infection and malignant transformation. In conclusion, the new perspectives and models in this study provide a working framework for investigating Treg within the self-reactive T-cell spectrum, expected to advance understanding of HTLV-1 infection, cancer, and immunotherapy strategies for these conditions.

## Graphical Abstract

**Graphical Abstract:**
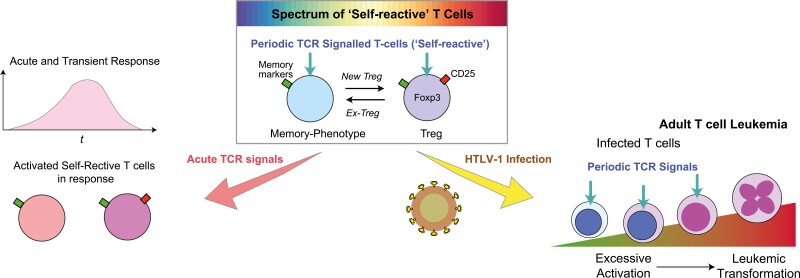

## Introduction

### Regulatory T-cells (Treg) and self-reactivity

Treg are conceptually defined as suppressive T cells and are experimentally identified as Foxp3-expressing CD4^+^ T cells [1, 2]. The majority of Treg in mice in a clean environment and in young people are considered to recognize an antigen in a tissue. This stems from the following three lines of evidence.

First, Treg differentiates in the thymus following recognizing a thymic antigen [3]. Medullary thymic epithelial cells can express tissue-restricted antigens in non-thymic tissues and thereby mediate T-cell selection processes, including negative selection for inducing apoptosis in self-reactive T cells and Treg selection to rescue them as Treg by inducing Foxp3 expression [4]. Some thymic CD4 single positive (SP) cells receive strong T-cell receptor (TCR) signals and may go through negative selection. However, after receiving strong TCR signals, some of the CD4 SP cells may express CD25, the interleukin-2 receptor alpha chain, and subsequently express Foxp3 only after persistent TCR engagements, becoming CD25^+^CD4 SP cells, which are identified as mature Treg cells [5].

The self-reactivity is also key to the homeostasis of Treg in the periphery. In the absence of TCR, Treg cannot maintain their number, as shown by Treg-specific TCR alpha knock-out strains [6, 7]. Indeed, Treg more frequently show cell division and proliferation than non-Treg *in vivo* and thus are dependent on homeostatic proliferation to maintain the size of their population [8].

Notably, while some conventional CD4^+^ T cells start to express Foxp3 in the periphery, most notably in intestines, after their exposure to microbiota [9, 10], such Foxp3^+^ Treg is also dependent on TCR signalling as Rag deficient mice are completely devoid of Foxp3^+^ Treg. These cells are designated as peripheral Treg, or pTreg, and may be enriched with T-cell clones different from Treg differentiated in the thymus.

The evidence above supports that Treg constitutes a mechanism to prevent T-cell activities against self-tissues in a broad sense, including tissue antigens and microbiota, by recognizing self- and innocuous antigens in the body. Thus, Treg is inherently ‘self-reactive’. Self-reactive T cells are defined by their ability to respond to host-derived antigens, including those from the body’s own tissues and commensal microbiota [11]. The presumed reactivity of Treg to ‘self’ makes them positioned in a unique role in cancer immunity and autoimmunity.

### The origin of Treg and memory-phenotype T cells

In addition to Treg, naturally occurring memory-phenotype T cells constitute another component of self-reactive T cells and are induced without direct immunization or infection. These two types of T-cell subsets constitute a spectrum of self-reactive T cells. The following lines of evidence support that Treg and memory-phenotype T cells constitute self-reactive T-cell populations.

First, both Treg and memory-phenotype T cells are dependent on the recognition of antigens *in vivo* for their differentiation. Importantly, while TCR transgenic mice with TCRs against an exogenous antigen harbour Treg and memory-phenotype T cells, both subsets are absent in a Rag-deficient background [12]. This indicates that the recombination of endogenous TCR genes allows some T-cells to produce TCRs reactive to some antigens in the thymus, promoting the development of Treg and memory-phenotype T cells. It should be noted, however, that transgenic TCR-expressing T-cells in a Rag-deficient background will acquire a memory phenotype after homeostatic expansion in a TCR signal-dependent manner [13].

Second, memory-phenotype T cells can generate Foxp3^+^ T cells more efficiently than naive T cells [14]. A fate-mapping study showed a considerable transition of cells into the memory-phenotype T cells from T cells that previously expressed Foxp3 [15]. In addition, Martin et al. showed that self-reactive conventional T cells are prone to express Foxp3 and become Treg [16]. Furthermore, the generation of memory-phenotype T-cells from the naïve T-cell pool is dependent on TCR signalling [17].

Third, memory-phenotype T cells may be enriched with cells that have the potential to regulate T-cell homeostasis. Ono et al. showed that depletion of GITR^high^ T cells, which lack CD45RB^low^ memory-phenotype T-cells, resulted in autoimmune inflammation across a broader range of organs compared to CD25^+^ depletion [18]. Recently, Whiteside et al. showed that some Foxp3- CD25^+^CD4^+^ T-cells with a memory phenotype can suppress T-cell activities in the absence of Foxp3^+^ Treg [19]. These memory-phenotype cells themselves exhibit capabilities to control the proliferation of naïve T cells, with potential mechanisms including clonal competition [20] and competition for cytokines [21].

The evidence above is not an exhaustive list of examples where the recognition of antigens in normal tissues, including microbiota, induces Foxp3 expression and promotes Treg differentiation. This leads to the feedback model of T-cell regulation, as per proposed by Ono and Tanaka 2016 [22], which suggests a dynamic interplay between Treg and memory-phenotype T cells. The model can be further updated by incorporating findings from Tocky systems as below.

### Periodic TCR-signalling in self-reactive T-cells revealed by Nr4a3-Tocky

While it is critical to understand the relationship between memory-phenotype T cells and Treg, the lack of a definitive marker transcription factor for memory-phenotype T cells has been a significant challenge. Moreover, all T cells exhibit a degree of ‘self-reactivity’, as they are selected in the thymus through recognizing self-peptide-MHC complexes (pMHC) [4]. These ‘weak’ interactions with pMHC are believed to be crucial not only for their development but also for their maintenance in the periphery [23–26]. Consequently, quantitative analysis of the dynamics of antigen recognition and the strengths of these interactions *in vivo* is required to address the self-reactivity of Treg and memory-phenotype T cells. Here, we propose the timer-of-cell-kinetics-and-activity (Tocky) tools as unique solutions, offering a novel approach to quantitatively investigate these critical aspects of T-cell biology.

**Figure 1:**
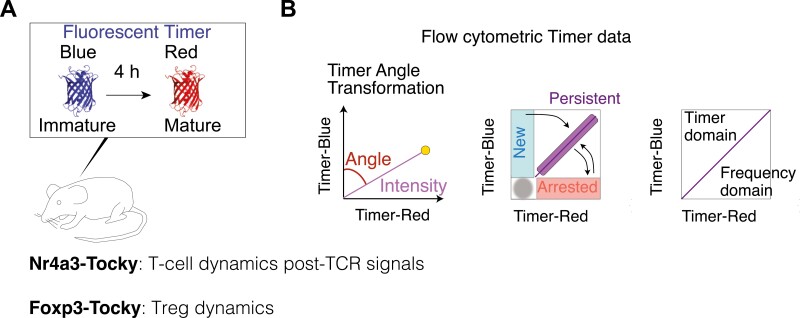
the Tocky system for analysing T-cell dynamics. (**A**) A schematic figure for Fluorescent Timer reporter mice (Tocky mice). Nr4a3-Tocky allows analysis of T-cell dynamics following TCR signals and Foxp3-Tocky enables investigation of Treg dynamics. The maturation half-life of immature blue and BFP-like fluorescence is 4 h and the protein half-life of the mature mCherry-type red protein is 120 h. (**B**) The Tocky locus approach and trigonometric transformation of Timer fluorescence data. Timer expression is converted into Timer Angle and Timer Intensity. The first half of Timer Angle (0˚–45˚) shows the temporal sequence of events following new transcription, while the second half (45˚– 90˚) represents transcriptional frequency, with values nearing 45° indicating the highest frequency

Nr4a3-Tocky is a transgenic mouse reporter model designed to analyse the temporal dynamics of T-cell activities following T-cell receptor (TCR) activation and cognate antigen signalling (Fig. 1A). In Nr4a3-Tocky, a fluorescent timer protein, fastFT [27], which emission spectrum matures from blue to red fluorescence with the maturation half-life of 4 h [28], is expressed in a synchronized manner to the Nr4a3 gene [5]. The mature red fluorescence protein has a long half-life, 120 h. Importantly, Nr4a3 is not expressed in resting T cells; however, its transient expression is highly induced upon TCR signalling within hours, and then rapidly decays [5]. Accordingly, the unique kinetics of the Fluorescent Timer protein and its maturation enable quantitative analysis of T-cell dynamics following TCR signals when appropriate algorithms and approaches are used. This forms the basis of the Nr4a3-Tocky system (Fig. 1B). The Tocky approach identifies three unique Timer loci: *New*, *Persistent*, and *Arrested*, which represent newly induced transcription, persistently sustained transcription, and once-activated-but-now-arrested transcription, respectively [5].

**Figure 2:**
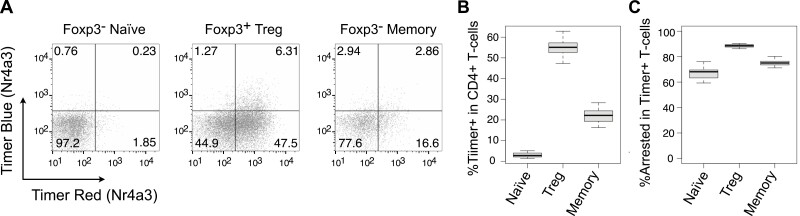
analysis of self-reactivity in CD4 + T-cells using Nr4a3-Tocky. (**A**) Flow cytometric analysis showing timer blue and red fluorescence across three CD4^+^ T-cell subsets from Nr4a3-Tocky mouse lymph nodes: CD44^low^ CD25^−^ Foxp3^−^ CD4^+^ T-cells (Foxp3^−^ Naïve), Foxp3^+^ Treg, and CD44^high^ CD25^−^ Foxp3^−^ CD4^+^ memory-phenotype T-cells (Foxp3^−^ memory). (**B–C**) Graphical representation of (B) the percentage of Timer-positive cells in CD4 + T-cells and (C) the proportion of cells in the Arrested locus among those Timer-positive CD4 + T-cells. The medians, along with the first and third interquartile ranges, are depicted by the bars. Sample size (*N*) = 7. Data were originally reported in [5]

Nr4a3-Tocky provides unique opportunities for identifying self-reactive T cells. In the Nr4a3-Tocky system, bulk CD4^+^ T cells comprise approximately 18% Timer-positive cells [5]. Foxp3^-^ CD4^+^ naïve T cells are mostly devoid of Timer^+^ cells, with only about 5% exhibiting Timer positivity (Fig. 2A and B). The most significant Timer expression is found in Treg, where over half of the cells are Timer-positive, suggesting a high enrichment of ‘self-reactive’ T cells within Treg. Furthermore, naturally arising memory-phenotype Foxp3^−^ CD4^+^ T cells contain about 20% timer-positive cells, indicating that a substantial fraction of memory-phenotype T cells are self-reactive.

Interestingly, irrespective of T-cell population, most of these Timer^+^ cells are Timer Blue-Red^+^ and are in the Arrested locus (Fig. 2C). This indicates that Treg and memory-phenotype T cells are composed of T-cells that have previously recognized antigen in the past several days but are not actively engaging with the antigen at the time of analysis [5]. This Timer expression pattern differs from that of T cells actively engaging with cognate antigen in inflamed tissue, which are primarily found in the Persistent locus [5]. These observations collectively demonstrate that Timer expression in the Nr4a3-Tocky system enables the identification of T cells that periodically recognize their cognate antigen and receive TCR signalling spontaneously under homeostatic conditions. We propose to designate these T-cells *Periodic TCR-signalled T-cells*, as a concept rooted in self-reactive T-cells but facilitating quantitative measurements and dynamic analysis.

The periodic TCR signalling is indeed essential for maintaining the Treg population during homeostasis. Notably, the number of Treg is reduced by approximately 2 weeks if they lack access to TCR signalling [6]. As the percentage of Foxp3-expressing cells is remarkably consistent under homeostasis within the same age or developmental stage [29, 30], the two lines of the evidence indicate that the homeostasis of the Treg population is regulated over days to weeks through Treg’s homeostatic proliferation.

### Foxp3 dynamics within periodic TCR-signalled T-cells

Foxp3 expression is dynamically regulated in both Treg and non-Treg CD4^+^ T-cells at the single-cell level. It is well-established that some Foxp3-expressing T-cells may lose Foxp3 expression both during homeostasis [15] and under inflammation [31], often designated as ex-Treg or ex-Foxp3 cells. Importantly, these ex-Treg cells usually have memory-phenotype or effector-phenotype.

To analyse the dynamics of Foxp3 expression, we developed the transgenic Foxp3-Tocky mouse strain, in which Fluorescent Timer protein expression is synchronized with the Foxp3 gene [5]. Importantly, using Foxp3-Tocky, we can clearly distinguish T cells that have recently initiated Foxp3 expression (i.e. new Treg) from those that express Foxp3 in a steady state (i.e. existing Treg). Interestingly, in non-inflammatory conditions, the majority of Treg exhibit intermittent Foxp3 expression between Persistent and Arrested loci. However, upon the induction of inflammation using a hapten-induced contact dermatitis model, some Foxp3 expressors upregulate and sustain Foxp3 expression over time, as identified in the Persistent locus [28]. Moreover, Foxp3 expression is newly induced upon the induction of inflammation.

### Two-axes of T-cell activation for Treg and memory-phenotype T-cells: periodic and acute TCR signalling

The periodic TCR signaling may not fully activate Treg, although it could be required for sustaining CD25 expression, which is a hallmark of Treg [6]. However, under inflammatory conditions, Treg can be activated, and once activated, Treg further increases the expression of ‘Treg markers’, including CD25, memory markers including CD44, and immune checkpoint molecules such as ICOS and CTLA-4. These activated Tregs are often designated as ‘*effector Treg’*. Since the high expression of CTLA-4 and other checkpoint molecules generally confer suppressive activities to T-cells, effector Treg is considered to have stronger suppressive activities [32], although this is yet to be directly demonstrated. Antigen recognition, mediated by TCR–pMHC interactions, is essential for T-cell-APC interactions as well as for Treg’s suppressive activities [33, 34]. However, the physical basis for T-cell–APC interactions extends beyond TCR-pMHC interactions to include additional adhesion and co-stimulatory interactions [35]. Notably, Treg, especially when activated, highly expresses costimulatory receptors such as ICOS, CTLA-4, and OX-40, and utilize adhesive molecules such as CD103 and LFA [36–38].

Importantly, effector Treg differentiation is promoted by the persistent transcription dynamics of Foxp3 expression, as revealed by Foxp3-Tocky [28]. These findings in mice are in agreement with the characteristics of effector Treg in humans, which are typically characterized as FOXP3^high^ CD25^high^ CD45RA^−^ CD4^+^ T cells [39], or as CD45RO^+^ instead of CD45RA^−^ [40]. In addition, effector Treg differentiation is dependent on Myb, IRF4, Blimp1, JunB, and RelA [38]. Signalling pathway candidates for effector Treg differentiation include IL-2 and TGF-β signalling, in addition to TCR signalling, as these pathways induce and enhance Foxp3 transcription.

Upon activation, it is probable that memory-phenotype T-cells acquire a phenotype akin to activated Treg, given that antigen-experienced CD4^+^ T-cell populations acquire highly activated and memory-phenotype upon activation [19, 41, 42]. In these instances, marker expression patterns, particularly the significant induction of CD25 expression upon activation, appear consistent. Nonetheless, the specific dynamics of memory-phenotype T cells during inflammation remain to be thoroughly explored and elucidated.

**Figure 3:**
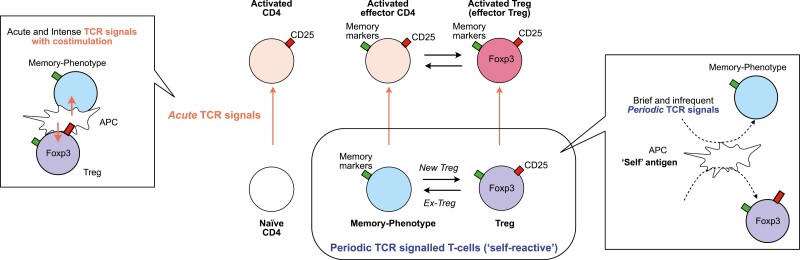
the two axes activation model for T-cell activation dynamics. This model introduces two dimensions of T-cell activation via T-cell receptor (TCR) signals: *Acute* and *Periodic* TCR signals, for CD4^+^ T cells, including naïve, memory-phenotype, and regulatory T cells (Treg). (1) Acute TCR signals: characterized by acute TCR signals accompanied by co-stimulation through CD28, facilitated by intense interactions with antigen-presenting cells (APCs), typically occurring during inflammation. These signals can activate all three T-cell populations, which are then identified as activated CD4 + T cells, activated effector T cells, and activated Treg (or effector Treg). (2) Periodic TCR signals: ‘self-reactive’ T cells, including Treg and naturally arising memory-phenotype T cells, periodically receive TCR signals through their ‘self-reactive’ TCRs, potentially recognizing self-antigens and innocuous antigens, such as those from microbiota. The interactions between T cells and APCs that induce periodic TCR signals are typically brief and transient. Note that some memory-phenotype T cells may initiate new Foxp3 expression and are then identified as Treg, while some Treg may lose Foxp3 expression and are then identified as memory-phenotype T cells

Here we propose a novel model for the regulation of Periodic TCR-signalled T cells, wherein Foxp3 expression within this subset is dynamically modulated, categorizing individual T-cells as either Treg or activated/memory-phenotype T cells based on observed Foxp3 levels (Fig. 3). Antigen stimulation exceeding the normal *Periodic TCR signals*, presumably with costimulation through CD28, triggers full T-cell-activation and induces the high expression of CD25 and other molecules during inflammation, irrespective of the dynamics of Foxp3 expression. This intense form of TCR signals, distinct from Periodic TCR signals, is termed *Acute TCR signals*. Consequently, our model delineates two principal activation axes for T-cells, in which Treg and memory-phenotype T-cells show unique dynamics. Although these two axes can influence Foxp3 expression dynamics, other factors such as IL-2 and TGF-β signalling and Foxp3 epigenetic modifications play significant roles. This model facilitates the operational definition of distinct T-cell clusters at the single-cell level, as elucidated later.

### The link between Treg studies and Human T-cell Leukemia Virus Type 1 (HTLV-1) research

HTLV-1 is endemic to a limited range of regions in the world, notably including South Japan, Caribbean countries, and parts of Africa [43]. Similar to its associated retrovirus, HIV-1 and HIV-2, HTLV-1 specifically infects CD4^+^ T cells via similar infection routes but does not induce T-cell apoptosis nor CD4^+^ T-cell reduction. Instead, a small percentage of HTLV-1 carriers experience the transformation of infected CD4^+^ T cells, developing unique and fatal leukaemia, Adult T-cell leukaemia/lymphoma (ATL) [44]. Some other patients may develop neuroinflammation, HTLV-1 associated myelopathy/tropical spastic paraparesis (HAM/TSP) [45, 46].

Studies on HTLV-1 and ATL played important roles in developing the materials and identifying key molecules that were required for the discovery and understanding of Treg. Currently, it is widely recognized that the concept of Treg was established by the two branches of studies, adoptive transfer experiments using lymphopenic animals, and genetic studies on Foxp3 [22]. Importantly, ATL cells were used as the material to clone the IL-2 receptor alpha chain gene, or CD25 [47]. As ATL cells cannot be cultured without the addition of a high dose of IL-2, Nikaido et al. hypothesized that the cells expressed IL-2 receptors in their cloning study. Here the establishment of an IL-2 receptor-specific monoclonal antibody (anti-Tac antibody), which used a cell line from ATL patients as an immunogen, led to the successful cloning of the IL-2 alpha chain, CD25 [47, 48]. The authors showed that normal T cells highly expressed CD25 upon stimulation, while ATL cells constitutively and highly expressed CD25. The discovery of the gene was soon followed by the development of CD25 KO mice, which develop autoimmune inflammation [49], establishing the role of CD25 and IL-2 signalling in immune regulation.

Meanwhile, coincidentally, the use of anti-CD25 antibodies in adoptive transfer experiments demonstrated the suppressive activities of CD25^+^ CD4^+^ T cells *in vivo*, defining Treg [50]. In humans, CD25^+^ CD4^+^ T cells include activated non-Treg T cells. Thus, Treg is defined by surface markers as CD25 high expressors (CD25^high^ CD4^+^ T cells) [51], CD25 expressors with class II HLA expression (CD25^+^ HLA-DR^+^ CD4^+^ T cells) [52], or CD25 high expressors with low IL-7 receptor expression (CD25^high^ IL7R^low^) [53].

Importantly the majority of ATL cells express CD25, identified as CD25^+^ CD4^+^ T cells [54], while FOXP3 expression is various [55, 56]. The similarity of the phenotype to the Treg one led to the speculation that ATL cells are the tumour of Treg cells. Whether ATL cells show suppressive activities like Treg is a matter of debate: while some studies show Treg-like activity in ATL cells [57], this notion is negated by the lack of suppressive activities in other reports [55, 58, 59]. As CD25 expression is induced in any T cells upon activation [60] and sustained under the presence of the HTLV-1 viral protein Tax [61], these evidence collectively support that HTLV-1 targets the activation mechanism in T cells that uses CD25 and IL2 signalling.

Furthermore, Satou et al. showed that the transgenic strain of HTLV-1 bZIP factor (HBZ), an anti-sense viral gene of HTLV-1, promoted the development of Foxp3^+^ T-cell lymphoma [62]. Foxp3^+^ CD4^+^ T cells start to be dramatically increased in adult mice, eventually transforming into lymphoma cells. HBZ is a transcription factor and not only induces Foxp3 expression but also physically interacts with Foxp3 and NFAT. This suggests that HBZ may interfere with the autoregulatory loop of Foxp3, in which Foxp3 protein binds to the gene regulatory region of the Foxp3 gene enhances Foxp3 transcriptional activities [28]. Intriguingly, the expression levels of Foxp3 in HBZ transgenic mice are lower than those of Treg in normal mice [63], suggesting that the HBZ-induced transcriptional machineries for Foxp3 are less efficient and less stable than the autoregulatory loop established in normal Treg.

### Activation machineries in self-reactive CD4^+^ T cells as a potential target of HTLV-1

**Figure 4:**
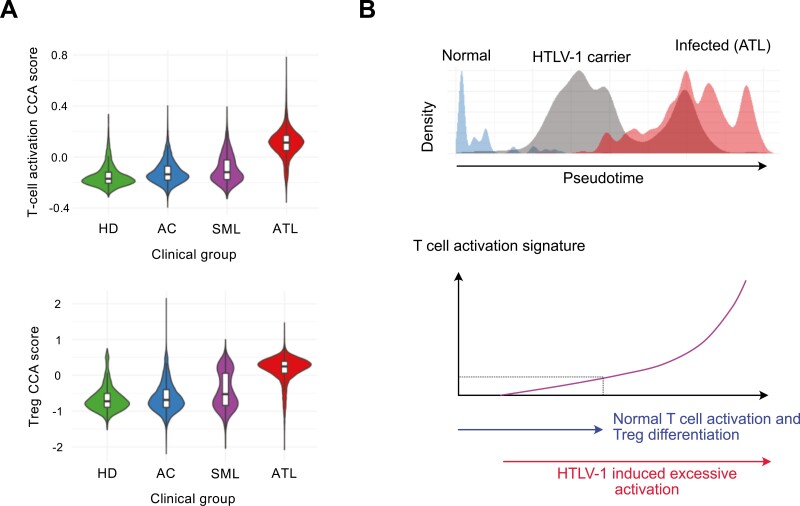
activation mechanisms in self-reactive CD4 + T-cells as potential targets of HTLV-1. CD4^+^ T-cells from both HTLV-1 infected individuals and healthy donors were analysed using single-cell RNA-seq. (**A**) Violin plots displaying activation signatures of single cells, analysed by canonical correspondence analysis (CCA) for T-cell activation (upper) and regulatory T-cell characteristics (Treg). HD denotes healthy donors, AC stands for asymptomatic carriers, and SML represents smouldering acute T-cell leukemia (ATL). (**B**) The cell distributions of the three categories, normal, HTLV-1 carrier (AC), and infected ATL, are shown along the trajectory for the progression of HTLV-1 infection and ATL leukemogenesis (pseudotime, upper). The lower panel shows a schematic representation of the dynamics of the CCA T-cell activation signature across the normal and infected individuals. Note that the activation dynamics across T-cells in normal individuals ("Normal T cell activation and Treg differentiation") are overlapped and followed by those in HTLV-1 infected individuals ("HTLV-1 induced excessive activation"), based on CCA activation analysis. This demonstrates that the excessive activation in HTLV-1 infected individuals can be extrapolated by the normal activation progression. The original data are presented in [51].

Recently Tan et al. made a key discovery to reconcile the controversies regarding HTLV-1 and Treg using single-cell analysis of CD4^+^ T cells from HTLV-1-infected people and patients with ATL [64]. They demonstrated that activation gene signatures are progressively induced and enhanced in HTLV-1-infected CD4^+^ T cells, directing them into a unique differentiation pathway. FOXP3, CD25, CTLA-4, and class II HLA, especially HLA-DR, are induced in if not all, significant percentages of cells (Fig. 4A). The progressive increase of these Treg markers supports that HTLV-1 infection and transformation exploits the activation machineries that can differentiate Treg.

Using canonical correspondence analysis (CCA) of transcriptomes [65], Tan et al. showed progressive enhancement of T-cell activation in CD4^+^ T cells from HTLV-1 carriers in comparison to healthy donors [64]. The enhanced activation was further aggravated and became excessive in ATL cells, exaggerating the effector Treg phenotype. Thus, HTLV-1-induced activation and transformation have been captured as a trajectory of cells and a continuous series of events [64]. Meanwhile, a genomic study showed that ATL cells accumulate mutations in genes related to TCR signalling, STAT3, and Notch pathways [66, 67].

Given the extremely long history of carriers developing leukaemia, which can span five to six decades, the precise mechanism for the moment of transformation is difficult to be directly analysed and demonstrated. However, the lines of the evidence above collectively indicate the important fact that the T cells that eventually transform into leukaemia cells must have opportunities to receive TCR signalling. Given the association of HTLV-1 and ATL with the Treg phenotype including FOXP3, CD25, CTLA-4, and HLA-DR, it is well possible that Periodic TCR-signalled T-cells, including Treg and memory-phenotype T-cells, are prone to the infection as well as the transformation due to their episodic TCR signalling and homeostatic proliferation (Fig. 4B). The prolonged period of slow and infrequent T-cell proliferation and activation may provide opportunities for the infected cells to accumulate mutations over decades, especially in the presence of genotoxic stress and transient expression of another oncogenic viral protein Tax [68, 69]. The interaction between HBZ and Foxp3 may direct the activation machineries towards Treg differentiation, albeit imperfect, as HBZ inhibits the expression of key functional Treg markers including CD25 and CTLA-4. This further supports the notion that Foxp3^+^ T-cells have defective functions in ATL [62]. Nevertheless, integrating the perspective of the self-reactive T-cell populations is essential for understanding the roles of T-cell activation machineries in HTLV-1 infection and transformation.

### Immunosuppression mechanisms in ATL and HTLV-1 infection

Intriguingly, ATL patients can have severe immunosuppression without the reduction of CD4^+^ T-cells [70]. This is not specific to malignant transformation, as HTLV-1 carriers can develop a significant immune suppression with difficult opportunistic infections even prior to the development of malignant disease [71, 72]. Importantly, HTLV-1 carriers show the reactivation of Epstein-Barr virus, a sign of immunosuppression [73] and have reduced naïve T-cells, whereas their memory-phenotype T-cell numbers are unaffected [74].

The underlying mechanisms for immune suppression in HTLV-1 are still unclear. HBZ transgenic mice show a marked increase of Foxp3^+^ T-cells [62], as discussed above, and intriguingly, are susceptible to herpes simplex virus infection [75]. However, it should be noted that the depletion of Treg rather worsens infections using the same virus infection model [76], suggesting that the weakened T-cell immunity by HBZ may be due to factors other than increased Foxp3 expression. Notably, HBZ suppresses activities of TCR signal downstream transcription factors, including AP-1 [77] and NF-kB [78], and represses cytokine expression, such as interferon-gamma (IFN-γ) [75].

Furthermore, single-cell analysis of PBMCs from HTLV-1 carriers and ATL patients by Tan et al. provides a unique perspective. It reveals that a substantial subset of CD4^+^ T-cells is chronically compelled to express T-cell activation machinery and immunoregulatory molecules such as CTLA-4 [64]. This chronic activation likely disrupts the T-cell system’s ability to coordinate its components and other immune cells effectively, potentially compromising the response to opportunistic infections. In addition, HTLV-1 infected T-cells express Class II HLA molecules [64], akin to normal CD25^high^ HLA-DR^+^ Treg [79]. Tan et al. demonstrated that Class II HLA was indeed functional in presenting antigens to other T-cells, which subsequently led to the induction of anergy-related genes in these antigen-presented T cells [64]. Future analyses of how HTLV-1 infection disrupts the behaviours of the T-cell system while aberrantly activating T-cells will further elucidate this intricate pathological mechanism in the retroviral infection.

### Dynamic perspective of periodic TCR-signalled T-cells in disease contexts

Lastly, we explore the application of the dynamic perspective of Periodic TCR-signalled T-cells in disease contexts beyond the ATL/HTLV-1 fields.

#### Tumour immunity

The dogma that Treg accumulation in tumours inhibits tumour immunity is widely accepted, yet emerging evidence suggests it may need to be reconsidered. While Tregs have been shown to suppress tumour immunity—a conclusion supported by the enhanced anti-tumour immunity observed following the depletion of the entire Treg population [80]. However, the relationship between Treg percentage and prognosis varies between studies [81–83]. Meanwhile, quantifying the contribution of Treg increases to tumour immunity suppression is challenging, partly because measuring the suppressive activity of individual Tregs *in vivo* is currently not feasible. In addition, the roles of memory-phenotype T-cells in cancer are still unclear, primarily due to difficulties in analysing Foxp3 dynamics across different T-cell populations and in distinguishing between predominantly self-reactive T-cells and cancer-reactive T-cells. This section discusses Treg evidence in cancer to elucidate current challenges and how the framework of Periodic TCR-signalled T-cells could benefit future studies.

Foxp3^+^ Treg accumulates in some tumours, including melanoma [40, 84]. The self-reactivity of Treg provides a unique insight, given that cancer cells are transformed self-tissue cells. Consequently, T cells reactive to an antigen in the cancer tissue are likely enriched with self-reactive T cells, the implications which remain to be fully clarified. Cancer-induced tissue destruction or chronic inflammation [85] may enhance the efficiency of APCs in presenting tissue antigens of normal cells, increasing the likelihood that Treg, rather than naïve T-cells, recognize antigens and receive TCR signalling. In addition, it has been reported that some patients with melanoma or breast cancers had tumours expressing Class II MHC (MHC II), possibly due to CIITA expression [86–88]. These findings, suggesting a potential impact on immune checkpoint blockade responses or clinical prognosis, warrant further investigation to determine the physiological significance of MHC II expression in tumours.

The action mechanism of checkpoint blockade on Tregs and memory-phenotype T-cells is not fully understood. Studies have shown conflicting results regarding whether anti-CTLA-4 antibodies deplete Tregs [89–92]. As CTLA-4 is highly expressed in Treg, especially in activated Treg [38], anti-CTLA-4 antibodies might predominantly affect these cells rather than naïve or non-cancer reactive T cells. Marangoni et al. showed that CTLA-4 blockade facilitated the proliferation of Treg in tumour, potentially negating immunotherapy benefits [93]. This study highlights the importance to investigate dynamic T-cell response to each immunotherapy and understanding feedback mechanisms. In addition, CTLA-4 on non-Treg cells can mediate its negative regulatory action on other T cells [94], although the effects of anti-CTLA-4 antibodies on these cells are even less clear. The expression of checkpoint molecules like CTLA-4 and PD-1, not typically induced by Periodic TCR signalling in circulation, suggests that the tumour microenvironment plays a crucial role in their expression. Here, Nr4a3-Tocky may provide unique insights into the two axes of T-cell activation and how they control susceptibility to the checkpoint blockade, while Foxp3-Tocky provides a coherent analysis of Foxp3 expression dynamics across different T-cell populations.

Some FOXP3^+^ T cells may function as effector T cells, producing pro-inflammatory cytokines in both healthy individuals [39] and melanoma patients [95]. This is linked to the level of FOXP3 expression: cells with high Foxp3 levels are enriched with functional Treg, whereas those with low to intermediate levels of FOXP3 expression include non-Treg effector T-cells that produce pro-inflammatory cytokines [96]. However, the absence of a distinct threshold for FOXP3 expression that can differentiate between non-Treg effector T-cells and functional Tregs complicates the identification of suppressive versus potential effector T cells [40]. Consequently, FOXP3 expression, when detected by anti-Foxp3 antibody, cannot serve as a definitive marker for suppressive activity. This challenge calls for an *in vivo* single-cell level analysis of Treg function. The Foxp3-Tocky tool emerges as a unique solution, enabling ‘real-time’ analysis of Foxp3 transcriptional activity to identify ‘persistent Foxp3 expressors’, which show effector Treg differentiation.

In conclusion, although Treg in cancer has been extensively investigated, the full immunological impact of cancer-reactive Treg remains elusive, hindered by technical limitations and the predominant approach to classify Treg vs non-Treg, which is becoming outdated and less helpful in the era of single-cell analysis. These identified challenges underscore the inadequacy of population-based methods and highlight the need for single-cell level FOXP3 expression and TCR signal dynamics analysis. Additionally, analysing the TCR repertoire of self-reactive T-cell populations, including Tregs and memory-phenotype T cells, is expected to shed light on CD4^+^ T-cell dynamics. Ultimately, exploring the spectrum across Tregs and memory-phenotype T-cells, particularly through the lens of the two axes of TCR signalling, acute and Periodic TCR signals, promises to deepen our understanding of CD4^+^ T-cell-mediated cancer immunity and the regulatory mechanisms of Foxp3 and TCR signals in CD4^+^ T-cell activities.

#### Autoimmunity

The self-reactivity of Treg aligns with their role in preventing autoimmune reactions. While this view is valid in Foxp3 mutation-induced autoimmunity in IPEX [97], Treg numbers and frequencies are various in autoimmune diseases in general, including multiple sclerosis [98] and systemic lupus erythematosus [99]. The role of Treg in those diseases is still controversial, even after nearly three decades of intensive study.

It is noted that the fundamental aspects of Treg theories in autoimmunity have been constructed based on results from Treg suppression assays, but the limitations of these methods should be acknowledged. While Treg’s suppressive activities can be assessed by bulk T-cell analysis using *in vitro* Treg suppression assays [100, 101] or certain autoimmune and inflammation models [85, 102, 103], these methods generally do not address key research questions regarding which subtypes of Treg are functional and suppressive. Additionally, outcomes by the most common protocols for *in vitro* Treg suppression assay are largely determined by two factors: the expression level of CD25 in Treg, which can absorb IL-2 molecules in culture, and the extent to which Treg is hyporesponsive to TCR stimulation in the culture [104].

Similar to the cancer context as discussed above, a significant challenge is that some FOXP3 expressors can produce proinflammatory cytokines in patients with autoimmune disease [105], similar to cancer patients. The analysis of Foxp3 dynamics within Treg and across Treg and non-Treg populations is a central issue, which further indicates the need of transition from population studies to single-cell level analysis. As discussed above, Foxp3-Tocky allows quantitatively analysis of Foxp3 transcription dynamics. Advancements in TCR-sequencing technologies are enabling quantitative analysis of TCR-repertoire overlaps between different T-cell populations or statuses. In the context of autoimmunity and immunotherapy, it is important to determine Foxp3 transcriptional dynamics in individual Periodic TCR signalled T-cells during under autoimmune inflammation and after treatment.

### Perspective beyond Treg: investigation of FOXP3 and TCR dynamics

The HTLV-1 studies highlight the biological and medical significance of the molecular mechanisms revolving around episodic TCR signalling and FOXP3 dynamic. Our studies and analyses support the model wherein HTLV-1 exploits for infection and transformation the capacity of Periodic TCR-signalled T-cells for episodically receiving TCR signals and occasionally yet more frequently proliferating than naïve T-cells. Thus, Periodic TCR-signalling, or self-reactivity, is considered the Achilles’ heel of the CD4^+^ T cell system, constituting a vulnerability to the virus infection and leukaemic transformation. This perspective is valuable not only for developing novel therapeutic strategies for HTLV-1 infection and ATL patients but also for improving the understanding of mechanisms for CD4^+^ T cells to control immunity. Given the vast diversity of T-cell repertoires in humans and mice, it is critically important to address regulations at the single-cell level or repertoire-level. Here our new dynamic perspective and the two axes of T-cell activation for Periodic TCR-signalled T-cells provide an operational framework for investigating CD4^+^ T-cell activities during homeostasis and disease conditions and for examining how cancer immunotherapy regimens work for controlling CD4^+^ T-cells at the single-cell level.

## Data Availability

The original experimental data in this study were previously reported as indicated in corresponding parts.
